# Effects of Exogenous and Endogenous Attention on Metacontrast Masking

**DOI:** 10.3390/vision2040039

**Published:** 2018-10-10

**Authors:** Sevda Agaoglu, Bruno Breitmeyer, Haluk Ogmen

**Affiliations:** 1 Department of Electrical and Computer Engineering, University of Houston, Houston, TX 77204-4005, USA; 2 Center for Neuroengineering & Cognitive Science, University of Houston, Houston, TX 77204-4005, USA; 3 Department of Psychology, University of Houston, Houston, TX 77204-5022, USA; 4 Department of Electrical and Computer Engineering, University of Denver, Denver, CO 80208, USA

**Keywords:** metacontrast, attention, exogenous attention, endogenous attention, visual masking, masking attention interactions

## Abstract

To efficiently use its finite resources, the visual system selects for further processing only a subset of the rich sensory information. Visual masking and spatial attention control the information transfer from visual sensory-memory to visual short-term memory. There is still a debate whether these two processes operate independently or interact, with empirical evidence supporting both arguments. However, recent studies pointed out that earlier studies showing significant interactions between common-onset masking and attention suffered from ceiling and/or floor effects. Our review of previous studies reporting metacontrast-attention interactions revealed similar artifacts. Therefore, we investigated metacontrast-attention interactions by using an experimental paradigm, in which ceiling/floor effects were avoided. We also examined whether metacontrast masking is differently influenced by endogenous and exogenous attention. We analyzed mean absolute-magnitude of response-errors and their statistical distribution. When targets are masked, our results support the hypothesis that manipulations of the levels of metacontrast and of endogenous/exogenous attention have largely independent effects. Moreover, statistical modeling of the distribution of response-errors suggests weak interactions modulating the probability of “guessing” behavior for some observers in both types of attention. Nevertheless, our data suggest that any joint effect of attention and metacontrast can be adequately explained by their independent and additive contributions.

## 1. Introduction

Visual masking is defined as the reduction in visibility of a (target) stimulus by another (mask) stimulus when they are presented in spatiotemporal vicinity of each other [1,2]. The term masking function refers to a plot of target visibility as a function of Stimulus Onset Asynchrony (SOA). Various types of masking have been identified based on spatial and temporal properties of the target and mask. Specifically, *metacontrast masking* occurs when a target is followed in time by a spatially non-overlapping mask. Visual masking plays a crucial role in information processing. It can suppress the contents of sensory memory, and thereby (i) eliminate motion blur and establish the clarity of vision for moving objects [3,4,5,6], and (ii) control the information transfer from sensory memory to visual short-term memory (VSTM) [7,8].

The visual system is flooded with an enormous amount of information under normal viewing conditions. Only a subset of this information can be selected for further processing. Attentional mechanisms are responsible for enhancing the processing of the selected information (features, objects, etc.) and for suppressing (or filtering out) the rest by allocating the available processing resources accordingly (e.g., References [9,10,11,12]). Attentional mechanisms are also involved in the maintenance of information in VSTM [13,14,15,16,17]. Since both attention and masking play a crucial role in the transfer of information from sensory memory to VSTM, it is important to determine whether these two processes interact or operate independently. 

Several studies reported interactions between attention and different types of masking (common-onset masking [18,19]; metacontrast masking [20,21,22]. However, most of these studies suffered from the methodological problems posed by ceiling and floor effects affecting measures of target visibility. Hence, whether the empirically observed interactions between attention and masking are merely side-effects of these ceiling/floor effects remains to be determined. Recent studies on common-onset masking reported no interaction between the two processes when ceiling and floor effects were avoided [23,24]. The relationship between attention and metacontrast masking in the absence of such artifacts remains to be established.

Two distinct types of attentional orienting have been identified [25,26,27,28,29,30,31]: (1) Exogenous attention has often been described as controlled by the stimulus and, therefore referred to as a reflexive mechanism. When we hear a loud bang or see a flash of light on a dark road, the visual system automatically deploys additional resources for processing this information [32,33,34]. (2) Endogenous attention, on the other hand, is a voluntary, rather than reflexive, allocation of resources to a predetermined region in space, a particular feature, or an object.

Peripheral cues (presented at or around the target stimulus) and central cues (generally presented at or near fovea) are generally used to activate exogenous and endogenous attention, respectively. Peripheral cues directly specify the target location and reflexively summon attention whereas central cues are conceptual in the sense that they need to be cognitively processed and interpreted to determine where and how to voluntarily deploy attentional resources. Due to these differences, exogenous attention reaches its maximum effectiveness at shorter cue-target onset asynchronies (CTOA) (100–120 ms depending on the task and the stimuli) compared to endogenous attention, which may require about 300 ms to reach its maximum effectiveness (e.g., References [35,36]). Moreover, exogenous attention effects decrease and disappear completely after 300–400 ms whereas endogenous attention benefits show a monotonically increasing trend as a function of CTOA and can be maintained as long as needed for the task [25,28,29,37]; see review in Reference [38]. Due to these differences in time courses, exogenous and endogenous attention are also called transient and sustained attention respectively [26,27,29,33]. However, let us highlight that, whereas exogenous attention is transient, endogenous attention is under voluntary control and can be transient or sustained depending on task demands. Hence, transient vs sustained distinction does not map uniquely to exogenous and endogenous attention.

Endogenous and exogenous attention can lead to similar perceptual changes (e.g., Reference [39]). Both types of attention have been shown to increase spatial resolution or acuity [40,41,42] and reduce temporal resolution [43,44], at the attended location. Suzuki and Cavanagh [45] showed that both types of attention distort the representation of position at the attended location. On the other hand, they can also lead to distinct perceptual effects. The effect of exogenous attention on conjunction search (based on the conjunction of multiple features) is larger than on simple search (based on a single feature) whereas endogenous attention yields equivalent improvements [46]. Dosher and Lu, in a series of studies, suggested that endogenous attention operates only under high-noise conditions whereas the benefits of exogenous attention can be found under both low- and high-noise conditions [47,48,49,50]. Ling and Carrasco [35], however, showed that both types of attention increase contrast sensitivity in both low- and high-noise conditions. Moreover, the modulation of contrast and response gains of neuronal responses have been associated with endogenous and exogenous attention, respectively [51,52]. Due to both differences and similarities between their temporal dynamics and between the perceptual changes they produce, there seems to be no consensus about the underlying neural mechanisms of these two types of attention. The view that the neural networks underlying endogenous and exogenous attention overlap to some extent, but are independent, has been supported by many studies [38] with one exception. Peelen, Heslenfeld and Theeuwes [53] used both central and peripheral cues in a functional neuroimaging study and reported that the same large-scale neural network mediates both types of attention. Nevertheless, whether masking has the same relationship with these two types of attention remains to be established empirically.

Attentional allocation of resources can also be controlled by changing set-size rather than by a spatial cue. In fact, in a recent study, we investigated the relationship between attention and metacontrast masking by varying set-size [54,55]. We presented an array of oriented bars around a virtual circle, and asked observers to report the orientation of a target bar, which was followed by an annulus with various onset asynchronies. We manipulated the attentional load by varying the number of bars in the display. We found that masking functions (i.e., performance as a function of target-mask stimulus onset asynchrony (SOA)) underwent uniform shifts of performance as set-size changed, suggesting that attention and metacontrast masking operate independently, an observation which was also supported by statistical analysis [54,55]. There are at least three limitations of controlling attention by set-size. First, several researchers suggested that the set size effect does not necessarily reflect attentional processing [56,57]. Assuming attentional processing capacity is limited, the rationale for using set-size is to tax attentional processes by increasing the number of inputs to be processed. In search experiments, performance is plotted as a function of set-size and the capacity of attentional processes is estimated by the slope of the performance-curve as a function of set-size. However, if we assume that inputs are noisy, it is shown that same results can be obtained by a low-threshold detection/discrimination model without assuming additional attentional capacity-limits. In a low-threshold model, noise associated with one or more distractors can trigger false-alarm responses, which therefore degrade performance, due to noise as opposed to attentional limits (see the review in Reference [57]). Second, varying set size does not allow us to investigate the *temporal dynamics* of attentional benefits. Third, since observers had to attend to the entire display at the beginning of each trial and the target was indicated by the onset of the mask, the task employs both endogenous and exogenous attention. Due to different temporal dynamics of the two types of attention and the aforementioned similarities and differences between the two, one cannot tease apart their contributions to performance. Differential contributions from different attention mechanisms might have overshadowed a potential interaction between metacontrast masking and attention in our previous study [54,55]. Here, we investigated the relationship between metacontrast masking and these two different types of attention separately by presenting either central or peripheral cues in different trial blocks. Our stimuli were positioned peripherally and hence a peripheral cue near a potential target serves as a natural way of attracting exogenous attention to that spatial location. If we were to use peripheral cues for endogenous attention, the same cue would attract both exogenous (due to its reflexive nature) and endogenous attention. Hence, to minimize selective effects of exogenous attention, the cue for endogenous attention was placed centrally so that it remained equi-distant from target and distractors. This way, any reflexive shift of attention to this cue would affect the processing of all stimuli (target and distractors) relatively equally. In our previous study, the mask also acted as the cue for the item selected for report. In the present study, using cues that are independent of masks also allowed us to remove any potential confound that could have arisen by the dual roles played by the mask in our previous study. By adjusting the stimulus parameters, we made sure that the ceiling/floor artifacts were avoided for each and thus every observer. Finally, we adopted a statistical modeling approach to determine whether endogenous and exogenous attention give rise to similar changes in the distribution of response errors. Part of the data from the present study has been presented at a conference [58].

## 2. Materials and Methods

### 2.1. Participants

Six observers (three males, three females; age range from 24 to 32) took part in this study and four of them were naïve as to the purpose of the study. All participants had normal or corrected-to-normal vision and gave written informed consent before the experiments. All experiments were carried out in accordance with the Code of Ethics of the World Medical Association (Declaration of Helsinki). We followed a protocol approved by the University of Houston Committee for the Protection of Human Subjects.

### 2.2. Apparatus

Stimuli were created using the ViSaGe and VSG2/5 cards manufactured by Cambridge Research Systems. A 22-in. CRT monitor with a refresh rate of 100 Hz and a display resolution of 800 by 600 pixels was used to present the visual stimuli. Observers sat at a distance of 1 m from the display. To restrict head movements of the observer, a head/chin rest was used.

### 2.3. Stimuli and Procedures

**Figure 1:**
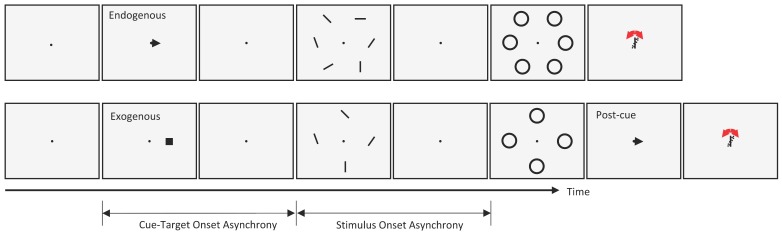
The stimulus sequences for both the endogenous (**top**) and exogenous (**bottom**) attention conditions.

To investigate the interactions between metacontrast masking and the two types of attention described above, we used either a central cue (endogenous attention experiment) or a peripheral cue (exogenous attention experiment) in separate trial blocks. The task of the observers was to report the orientation of a target bar whose location was indicated by a pre-cue at the beginning of each trial. The stimulus sequences for both cueing types are given in Figure 1. Each trial started with a fixation point on an otherwise blank gray (60 cd/m^2^) screen. After a random delay (500–1000 ms), a black pre-cue (an arrow at the center in the endogenous attention blocks, or a 0.3 deg square at 3.0 deg eccentricity in the exogenous attention blocks) was shown for 50 ms, indicating the target location. After a variable CTOA, an array of six (endogenous) or four (exogenous) oriented bars (1 deg long, 0.1 deg wide) was presented for 10 ms around an imaginary circle centered on the fixation point, so that all bars had the same retinal eccentricity. In the exogenous attention blocks, we had 4 oriented bars and the eccentricity of each bar was 5 deg. In the endogenous attention condition, we had 6 oriented bars. We increased the eccentricity for each bar to 6 deg so that the bars would not be too close to each other.

The main reason for using 4 instead of 6 oriented bars in the exogenous attention condition was to reduce the total number of trials for the experiment. Since the cue had to be non-informative across trials, all locations were cued with the same probability. With 4 items, 1200 trials out of 4800 corresponded to valid trials and were analyzed for the main results. Had we used 6 items as in endogenous attention experiment, we would have had to collect 7200 trials per observer to have the same number of valid trials. Since it was difficult to recruit observers who would participate in both experiments with a total of more than 7000 trials (1600 endogenous + 4800 exogenous + practice trials), we reduced the number of potential test bars in the exogenous attention experiment.

All oriented bars were followed by a metacontrast mask (a ring with inner and outer diameters of 1.1 deg and 1.4 deg, respectively) after a variable stimulus onset asynchrony (SOA). Typical metacontrast functions follow a U-shaped curve. In order to have results that are generalizable, we selected multiple SOA values that captured the U-shape of metacontrast functions. During metacontrast experiments, attention comes to play: Typically, observers will engage their endogenous attention to extract in an efficient way target-information based on task demands (for example, if the target location is predictable, then endogenous attention will be directed to the target location; if target location is distributed among multiple locations with equi-probabilities, then it is likely that observers will spread their attention to all potential target locations). The onsets of the target and mask will attract exogenous attention and the locus of exogenous attention *will depend on SOA*. Given that attentional processes are likely to depend on SOA, manipulations of attention are likely to affect attentional effects differently as a function of SOA. This would, in turn, result in interactions. Rejecting the interaction hypothesis requires stronger evidence when carried out on multiple SOAs compared to a single SOA.

Within the same block, the mask array was not presented in some trials, and the performance in these trials served as the baseline-performance level for each cueing condition. Once the stimulus sequence was presented, responses from observers were collected via a gamepad. Observers were asked to adjust the orientation of a comparison bar, by pressing right or left buttons on the gamepad, until a best match to the orientation to the previously presented test stimulus was obtained.

Even though central and peripheral cues are designed to guide endogenous and exogenous attention, respectively, the validity of each cue may also play a role in observers’ strategies on how to allocate their attentional resources. For example, if an endogenous cue is not 100% valid, observers may distribute some of their attentional resources to uncued locations so as to increase performance in invalid trials. For that reason, in the endogenous attention experiment, the central cue had 100% validity so as to maximize the voluntary allocation of resources to the location of the target bar. In the exogenous attention experiment, however, observers reported the orientation of the bar indicated by a second cue, i.e., a post-cue, which appeared at the end of each trial. The peripheral pre-cue in the exogenous attention condition was not informative of the target location, since the chance of being pre-cued was the same for all four targets (25% validity). Given that exogenous cues were uninformative, we would expect observers not to allocate endogenous-attention resources in a cue-dependent manner, thereby minimizing any potential contribution from the endogenous attention mechanisms in the exogenous-attention trials. Note that exogenous and endogenous attention conditions with different cue-validities are analyzed separately. There was no time limitation on the response, and observers initiated the next trial by another button press. In the endogenous attention experiment, three CTOA values (0, 200, and 500 ms) were used whereas in the exogenous attention experiment, only two CTOAs were used; 0 ms and a CTOA between 80 ms and 120 ms (specific values for each observer were as follows: 120 ms for ATB, 80 ms for EB, 100 ms for FG, 80 ms for GQ, 100 ms for MNA, and 80 ms for SA), where the effect of exogenous attention is largest, as determined by pilot studies. In both cueing conditions, five individually suited target-mask SOAs were used for each observer.

In both conditions, each block started and ended with 10 consecutive baseline trials. Moreover, different SOAs were interleaved in the remaining trials within a block. In each block, the same CTOA was used. In other words, cue timing was varied across blocks whereas target and mask timing was randomized within blocks. Each combination of CTOA and SOA values, as well as the baseline conditions were run 100 times. In total, each observer completed 1800 trials ([5 SOA + 1 baseline] × 3 CTOA = 18 conditions) in the endogenous attention experiment, and 1200 valid trials ([5 SOA + 1 baseline] × 2 CTOA = 12 conditions) out of roughly 4800 trials (i.e., 25% validity) in the exogenous attention experiment. 

To familiarize observers with the task and the experiment, and stabilize the effects of perceptual learning, we ran several practice blocks (<500 trials per observer) with all conditions before we started the actual experiments. Practice trials were not included in further statistical analyses. 

### 2.4. Avoiding Floor and Ceiling Effects

The target and mask luminances were adjusted for each observer to avoid floor/ceiling artifacts. For each observer, the floor is defined as the theoretical chance level, whereas the ceiling is defined empirically as the highest performance achieved when there is no mask (i.e., baseline performance). Target-mask luminance pairs were selected to satisfy simultaneously two criteria: (C1) To establish that performance did not suffer from a ceiling effect, the highest masked performance had to be significantly lower than unmasked (baseline) performance; and (C2) To establish that performance did not suffer from a floor effect, the lowest masked performance had to be significantly higher than chance level. Moreover, because (1) in metacontrast optimal masking occurs at positive target-mask SOAs and (2) observers can differ in the value of the optimal SOA, a separate SOA range was also suited to each observer.

### 2.5. Statistical Analyses and Modelling

#### 2.5.1. Performance Measures

(1) Statistical distribution of errors

We calculated signed response-errors as the difference between actual and reported orientations of the target bar, i.e., $Error\;Angle=\theta_{a}-\theta_{r},$ where $\theta_{a}$ and $\theta_{r}$ are the actual and reported angles, respectively. Response-error values ranged from −90 to 90 deg. As discussed in the Data Analysis section below, the distribution of signed response-errors was used for statistical mixture-modeling.

(2) Transformed performance

In order to obtain masking functions, the magnitude of signed response-errors, |Error Angle|, was transformed to a probability-like measure via Equation (1) [14]:

(1) $$Transformed\;Performance=1-\frac{\left|Error\;Angle\right|}{90}.$$

This is a *linear* transform of the magnitude of error angles to a probability-like range, where 0.5 and 1 correspond to chance and perfect performance, respectively. This allows an easier interpretation of the results. As discussed in the Data Analysis (Section 2.5.2) below, transformed performance was used to assess attention metacontrast interactions through metacontrast functions.

#### 2.5.2. Data analyses

We conducted four separate data analyses:

***Analysis (1) Avoiding floor and ceiling effects:*** For the first analysis, the goal was to establish that floor and ceiling effects are indeed avoided. For this purpose, we ran a power analysis to determine the number of trials per SOA at 0.7 power level based on the data from pilot experiments and our previous studies on metacontrast masking. Note that this value does not reflect the overall power of our other two analyses described below, nor the power of across-observers tests. It is merely used as an objective criterion to set *a priori* the number of trials per observer. This analysis yielded around 200 trials in total for baseline (i.e., no mask condition) and masking conditions. Hence, each observer (except GQ who ran 70 trials for both conditions) ran 100 trials per SOA for both masking and baseline conditions. Table 1 lists the target and mask luminances, as well as the results of *t*-tests used to check whether or not both criteria listed above were met, for all observers. In general, *p*-values indicate highly significant differences from ceiling and floor levels, indicating that floor and ceiling effects are avoided.

***Analysis (2) Assessing attention metacontrast interactions by analyzing masking functions:*** The second analysis was directed to masking functions with the goal of determining whether attention and metacontrast interact. Because we used different stimulus parameters for each observer for the reasons mentioned above, we adopted a within-observer analysis approach and analyzed transformed-performance of each observer separately. We fitted a series of linear and polynomial embedded regression models listed in Table 2 to determine the contributions of the main factors (e.g., CTOA, SOA) *and their interactions*. In the exogenous attention condition, only the trials where the peripheral cue correctly indicated the target location (i.e., valid trials) were included in the analyses. The results of the invalid trials in the exogenous attention condition were analyzed separately and included in Appendix A.

We used both Bayesian Information Criterion (BIC) and Adjusted-R^2^ metrics in the selection of best performing model. Model selection results were similar, if not identical, with both metrics for all observers. Both metrics penalize models for the number of free parameters. In addition, the BIC approach provides comparisons between different models in terms of their likelihood. To compare models, one needs to look at differences between BICs from different models. A BIC difference of x between model A and model B (i.e., BIC_A_−BIC_B_) corresponds to e^−x^-to-1 odds favoring model A. Therefore, the regression model with the smallest BIC value is the most likely model compared to others, and the BIC difference between two models indicate their relative likelihood.

***Analysis (3) Statistical mixture modeling of data:*** Statistical mixture models have a long history in behavioral, perceptual, and cognitive studies. In several studies, it was noted that a mixture of statistical models (e.g., combined Gaussian and Uniform distributions) provide a better account of data compared to a single one (e.g., Gaussian), even when the difference in the number of parameters is taken into account. Mixture models have been used in modeling VSTM [14,59,60,61], visual encoding [14,60], crowding [62,63], and masking [64,65]. An upshot of this approach is that it can provide a meaningful interpretation for the parameters of the model. For example, as discussed below, for a Gaussian + Uniform mixture model, the mean and variance of the Gaussian can be interpreted as the accuracy and precision of the underlying process, respectively, whereas the weight of the Uniform component can be interpreted as the guess rate.

We used a family of embedded statistical models: The G model has only a Gaussian term, and the GU model is a weighted sum of a Gaussian and a Uniform distribution. The GUCA and GUNN models have an additional Gaussian term, which represents “misbinding” behavior (i.e., reporting the orientation of a non-target bar). Since the non-target bars share structural properties of the target bar, there is a possibility to report one of the non-target bars, e.g., the one that has the closest angle to the target angle or the closest location to the target location, instead of the target stimulus. In this case, the masking effect would be caused by incorrect identity binding of a feature of a non-target bar to the target bar and this effect is modeled by an extra Gaussian distribution. In the GUCA model, misbinding is caused by the non-target bar, which has the closest angle to the target’s orientation. In the GUNN model, misbinding is modeled as stemming from the nearest neighbors of the target bar. Since the adjacent neighbors are equally far from the target bar, two non-target Gaussian distributions were included for the nearest elements in the GUNN model. We did not include all non-target elements for misbinding because, as discussed in Reference [60], including all elements can lead to spurious matches, which in turn can lead to an over-fitting of these misbinding terms. For more details, the reader is referred to References [60,64].

We used the Bayesian Model Comparison (BMC) technique [66,67] for selecting the best performing statistical model. The performance metric provided in Equation (2) will be referred to as the BMC (for full derivation see References [55,64]). We assumed uniform priors over a plausible range of parameters and calculated the BMC as

(2) $$\ln\;L(m)=\ln\;L_{max}(m)-\sum_{j}^{k}\ln\left(R_{j}\right)+\ln[\int exp(\ln L(m|\theta )-\ln L_{max}\left(m\right))d\theta ],$$

where *m* represents the models, θ represents the free parameters of the models, $R_{j}$ represents the size of the range for jth free parameter and *L_max_*($m_{j}$) = max(*L*($m_{j}$|*θ*)). We used the Riemann-sum approximation to compute Equation (2) and we had at least 50 bins in each parameter dimension. The difference between the BMC values from two different models is equivalent to the logarithm of their likelihood ratios. Therefore, a model with larger BMC performs better. A BMC difference of x between model A and model B corresponds to e^x^-to-1 odds favoring model A.

***Analysis (4) Analysis of winning statistical model’s parameters*:** Analysis 2 is conducted for determining whether attention and metacontrast interact. Analysis 3 provides a parametric interpretation of the data. We investigated the relationship between masking strength and model parameters by computing the correlation (Pearson R coefficients) between masking functions and the model parameters. The masking function is a plot of target visibility as a function of target-mask SOA. A strong correlation would suggest a critical role for that model parameter in accounting for masking effects, and a change in correlation with CTOA would suggest an interaction between attention and masking *for that parameter (or for the process represented by that parameter)*.

After model selection, we examined the winning model parameters to determine how model parameters, which represent different mechanisms (e.g., stimulus encoding, guessing), change with SOA and CTOA in endogenous and exogenous attention conditions. The examination of model parameters has the potential to tease apart different relationships between the metacontrast and attention processes they represent. We created 500 different data sets of response errors using resampling by replacement method for each and every observer separately. Then, we fitted the best model to these data sets. We obtained means and standard errors for model parameters of the winning model by this bootstrapping method. Finally, we fitted a series of linear and polynomial regression models (see Table 2) to each model *parameter* for each observer separately. By this, we were able to reveal the contributions of the main factors (e.g., SOA, CTOA) and their interactions for each model parameter.

## 3. Predictions

**Figure 2:**
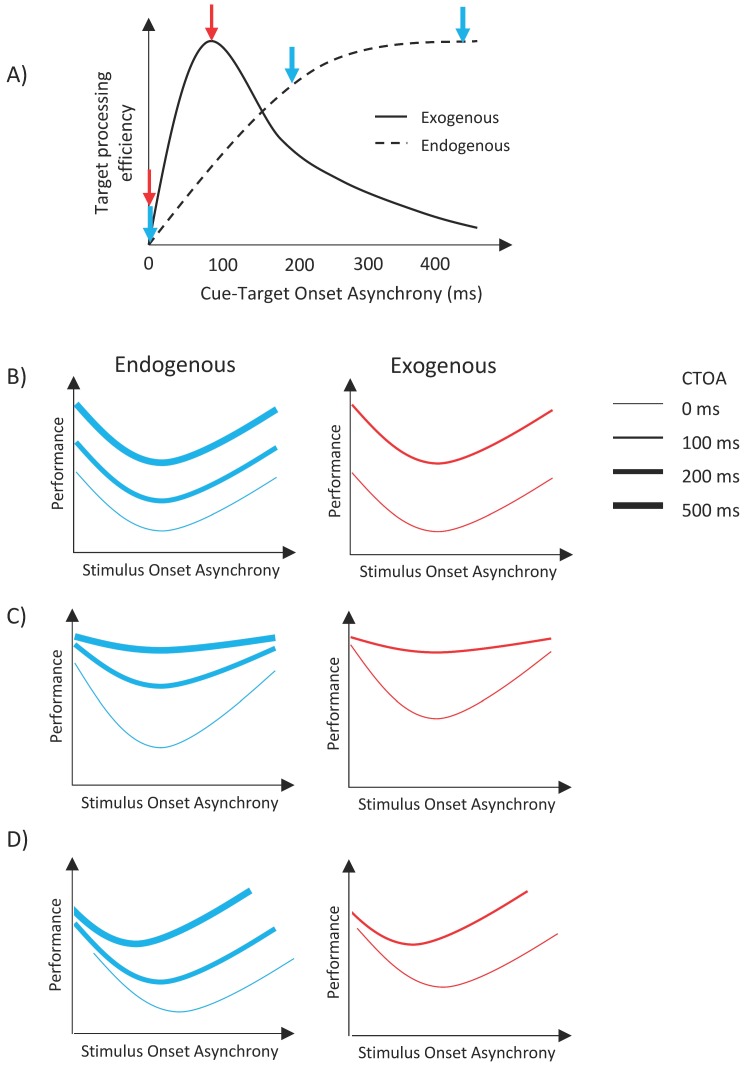
(**A**) The time courses of effects of exogenous (solid line) and endogenous (dashed line) cueing [68]. The blue and red arrows indicate endogenous and exogenous cues, respectively. (**B**) The predicted outcomes assuming no interaction between attention and masking. (**C**,**D**) Possible outcomes that would indicate interactions between metacontrast and attention.

As we mentioned in the Introduction, the enhancements of target processing with endogenous and exogenous orienting of attention have been known to have different time courses (see review in Reference [68]). Figure 2A illustrates the time courses and the predicted outcomes for the experiments presented here. When either type of cue is shown simultaneously with the target item (i.e., CTOA = 0 ms), both cues are ineffective; however, as the time separation between the cue and the target is increased, the facilitative effect of exogenous attention increases first, peaking around 100–120 ms, and then decreases back to no facilitation at long CTOAs [25,28,29,37]. For endogenous attention, the facilitative effect increases monotonically and reaches a plateau after a certain CTOA [25,28,29,37]. Here, we investigated whether different types of attentional orienting interact with metacontrast masking. If there is no interaction, then masking functions (i.e., transformed performance as a function of SOA) should uniformly shift vertically up or down, with the size of the shift depending on CTOA. Specifically, masking functions should shift upward with increasing CTOA for the case of endogenous cueing whereas it should shift up first, and then shift down to its no facilitation levels for exogenous cueing (see Figure 2B). However, since we used only two CTOAs in the exogenous attention condition, our data can only show an upward vertical shift from zero CTOA to 100 ms CTOA. On the other hand, any other pattern of results, i.e., any nonuniform change in masking functions, such as a change in maximum deviation in masking strength as a function of SOA with CTOA (Figure 2C), or a shift of the dip of the masking functions with CTOA (Figure 2D), or any combination of these two changes would indicate an interaction between attention and masking.

## 4. Results

**Figure 3:**
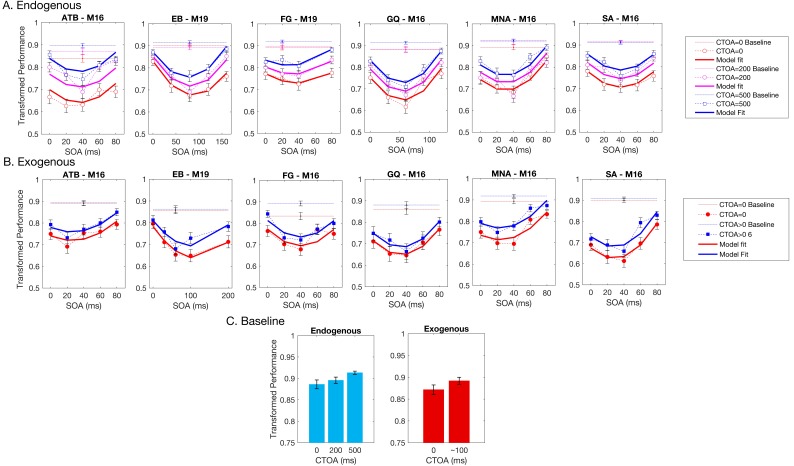
The transformed performance in the (**A**) endogenous and (**B**) exogenous attention conditions for all CTOAs and SOAs. The horizontal axes represent SOA and the vertical axes represent transformed performance (see Methods, Section 2). Different colors represent different CTOA conditions. The dotted horizontal lines indicate baseline (i.e., without masks) performance. The markers and the dashed lines represent empirical data, whereas the solid lines show the best-fit regression model. Each panel shows data from a single observer. The initials of each observer and the best regression model (see Table 2) are given on top of each panel. Error bars represent ± SEM across trials (n = 100). Note that only the validly cued trials are included in both conditions, which correspond to 100% and 25% of the trials in the endogenous and exogenous attention conditions, respectively. Results of invalidly cued trials in exogenous attention condition is shown in Appendix A. (**C**) The baseline performance (averaged across observers) as a function of CTOA in both conditions. Error bars represent ± SEM across observers (n = 6).

Figure 3 shows the experimental results for both cueing types obtained by each observer. The vertical axes represent the transformed performance while the horizontal axes represent SOA between the target and mask arrays. The dotted lines represent baseline conditions where the mask array was not presented. The markers and dashed lines represent empirical data whereas the solid lines indicate the best fitting regression models. Different colors represent different CTOAs. The baseline data were collected to ensure that the masking data did not have any ceiling effect (criterion C1, see Methods, Section 2). For each observer, we performed a two-sample *t*-test between the baseline and masking conditions at an SOA where masking is the weakest, i.e., the transformed performance is the highest. Moreover, we did a one-sample *t*-test between the chance level (0.5 transformed performance) and the masking conditions at an SOA and CTOA pair where the transformed performance is the lowest (typically, zero CTOA and an intermediate SOA), to ensure that floor effects are also avoided (criterion C2, see Methods, Section 2). Table 1 lists the results of all *t*-tests, as well as the target and mask luminances that allowed us to avoid ceiling and floor effects for each observer. In short, both criteria were met for all observers, and our masking data are free from ceiling and floor effects.

**Figure 4:**
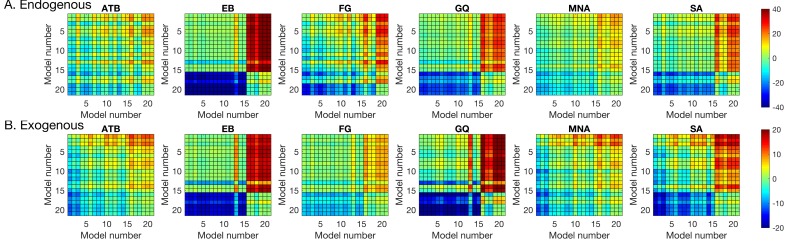
The Bayesian Information Criterion (BIC) differences between each pair of the regression models listed in Table 2. (**A**) Endogenous attention condition. (**B**) Exogenous attention condition. Greenish colors represent equivalent model performance whereas blue and red colors represent better and worse model performance, respectively, in comparing the model listed on the y axis to the model listed on the x axis.

With the possible exception of observer EB’s results, visual inspection of Figure 3 suggests a general trend, whereby masking functions seem to be shifted uniformly along the ordinate with changes in CTOA, consistent with the predictions of no interaction between masking and attention (cf. Figure 2). To quantitatively test this qualitative observation, we fitted a series of polynomial regression models (see Table 2) to individual data to quantify the effects of SOA (masking), CTOA (attention), and their various interactions. The best model was selected based on the BIC metric (the lower the BIC, the better the model), which pits model likelihoods against each other after taking into account the number of parameters. The pairwise BIC differences are given in Figure 4. Greenish colors represent equivalent model performance whereas blue and red colors represent better and worse model performance, respectively, in comparing the model listed on the y axis to the model listed on the x axis. Figure 3 also shows the best model fits (solid lines).

In the endogenous attention condition, for four out of six observers, the best model was M16. This model has linear SOA and CTOA terms, as well as a quadratic SOA term, but no interaction term. For observers EB and FG, the best model was M19, which has an additional interaction term. In the exogenous attention condition, for all observers except EB, M16 was again the best regression model. For EB, M19 again performed best. Note that although the best regression model was M19 for EB, the BIC differences between M16 and M19 were within ±2 indicating that these two models performed nearly equally well. Hence, five out of six observers indicate no interaction and the evidence for interaction in the sixth observer is very weak. Taken together, our results suggest that when targets are masked, manipulations of the levels of metacontrast and of endogenous/exogenous attention have largely independent effects. Our experiments were very time-intensive for our subjects, especially given that the parameters were needed to be adjusted individually for each subject to avoid ceiling and floor effects. Our results show some variation across subjects and stronger evidence for broad populations will require additional experiments, possibly using only a subset of experimental conditions on a very large number of subjects sampling adequately the targeted population.

Perceptual improvements as a result of spatial pre-cueing have been reported to be contingent upon the presence of masks (e.g., References [49,50,69,70,71]). To test whether the effect of cueing is limited to the cases where masks were presented in our experiments, we analyzed the transformed performance in the baseline conditions (see Figure 3C). Although we did not control for ceiling and floor effects in the baseline conditions, we found a significant improvement in transformed performance with increasing CTOA in the endogenous attention condition. A one-way repeated measures ANOVA yielded a significant main effect of CTOA (F_2,10_ = 8.060; *p* = 0.008; η_p_^2^ = 0.617). Although there was an increasing trend in performance with CTOA, a paired *t*-test between performance at zero CTOA and ~100 ms CTOA in the exogenous attention condition was only marginally significant (t(5) = 2.451; *p* = 0.058). 

### Statistical Mixture Modeling

**Figure 5:**
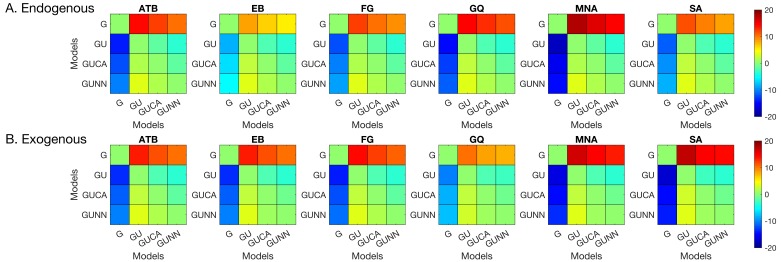
Pairwise Bayesian Model Comparison (BMC) differences between the statistical models tested. A square with coordinates (x,y) on each plot represents the BMC difference between model y and x. In order to have the same color notation (i.e., cooler colors mean better model performance and hotter colors mean worse model performance) as in Figure 4, we flipped the sign of the BMC differences. For both types of attention and for all observers, the GU model performs best in explaining the distribution of signed response errors, as indicated by the darkest blue color at the (G, GU) coordinate in all panels.

We examined the distribution of signed response-errors by using the BMC technique. We used a hierarchy of statistical models to capture the characteristics of the response errors (see Methods, Section 2). Among these models, the GU model was the winning model for all observers in both types of attention manipulations. The pairwise BMC differences are given in Figure 5. Averaged across observes, the BMC of the GU model in the endogenous attention condition was larger by 13.1, 1.9, and 3.4 than that of the G, GUCA, and GUNN models, respectively. These differences correspond to 5.0 × 10^5^-to-1, 6.7-to-1, and 30.0-to-1 odds, in favor of the GU model, and suggest a “decisive evidence” favoring the GU model [72]. Similarly, in the exogenous attention condition, the BMC of the GU model was 14.3, 2.1, and 3.2 larger than that of the G, GUCA, and GUNN models, respectively. These BMC differences correspond to 1.6 × 10^6^-to-1, 8.2-to-1, and 24.5-to-1 odds, all favoring the GU model. Next, we analyzed the model parameters of the GU model to determine whether any interaction between metacontrast masking and attention exists. Since the Gaussian and the Uniform components in the GU model are interpreted to represent different processes (stimulus encoding and guessing), examination of model parameters has the potential to tease apart different relationships between these processes, metacontrast, and attention.

**Figure 6:**
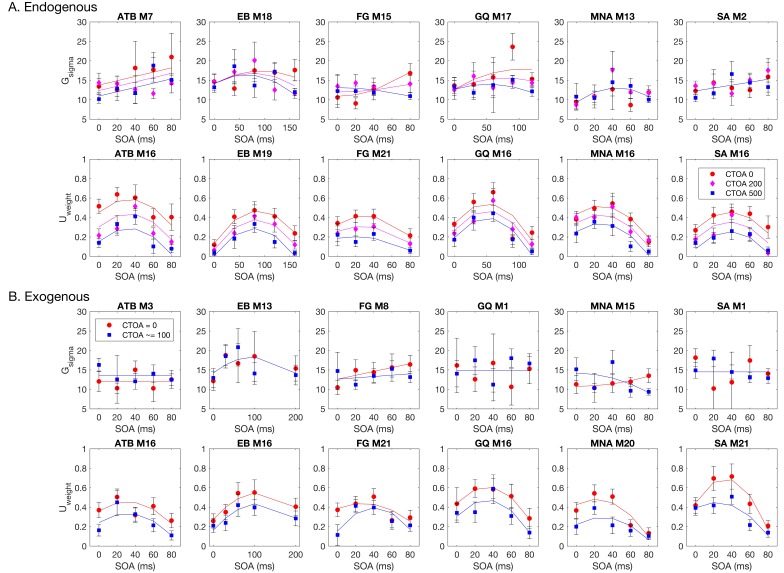
Pairwise BMC differences between the statistical models tested. A square with coordinates (x,y) on each plot represents the BMC difference between model y and x. In order to have the same color notation (i.e., cooler colors mean better model performance and hotter colors mean worse model performance) as in Figure 4, we flipped the sign of the BMC differences. For both types of attention and for all observers, the GU model performs best in explaining the distribution of signed response errors, as indicated by the darkest blue color at the (G, GU) coordinate in all panels.

Figure 6 shows the model parameters for the winning GU model as a function of SOA and CTOA in both the endogenous (Figure 6A) and exogenous (Figure 6B) attention conditions. Across observers, there is no clearly discernable systematic pattern of changes in the standard deviation of the Gaussian term. The weight of the Uniform component, however, depicts an entirely different picture. First, it tightly follows the (inverted) shape of masking functions, indicating that metacontrast masking exerts its effect primarily by increasing the weight of the Uniform component (i.e., guessing). Second, the effect of pre-cueing at different temporal distances to the target array (i.e., CTOAs) is also reflected in the weight parameter as an overall increase/decrease at all SOAs. At zero CTOA, where spatial pre-cueing virtually has no effect on performance, the weight parameter is largest for all SOAs and observers in both types of attention. As CTOA increases, more attentional resources are deployed at the target location, which decreases the weight of the Uniform component. More importantly, these opposing effects of metacontrast and attention seem to be operating independently, since the weight functions (i.e., the weight of the Uniform component as a function of SOA) undergo vertical shifts with CTOA.

These informal evaluations of the results were confirmed by the statistical tests where we fitted model parameters with a series of polynomial regression models. Figure 6 also shows the best fitting regression model on top of each panel. Across observers, there were differences in the regression model that best captures the changes in the standard deviation of the Gaussian term. These inconsistencies across observers suggests that masking strength and attentional benefits are not directly reflected in the standard deviation of the Gaussian in the GU model. The changes in the weight of the Uniform term were best captured by the regression model M16 for four out of six observers in the endogenous attention condition. For the observers EB and FG, the best regression model was M19 and M21, respectively. The model M19 has an additional SOAxCTOA interaction term compared to M16, and the model M21 has both SOAxCTOA and SOA^2^xCTOA interaction terms (see Table 2 for a complete list of all regression models). In the exogenous attention condition, the best regression model for the weight of the Uniform term was M16 for three out of six observers. Here, the best model for observer FG was again M21. However, for observer EB, there was no interaction between SOA and CTOA in the exogenous attention condition. Moreover, for observers MNA and SA, the best regression models were M20 and M21, respectively. Both M20 and M21 contain a quadratic SOA and CTOA interaction, which suggest a masking strength-dependent effect of attention. This is apparent in the nonuniform, SOA-dependent drops in the weight parameter with an increase in CTOA for these observers (Figure 6B, U_weight_). Interestingly, we did not find such interactions for the weight parameter in the endogenous attention condition, as well as the transformed performance in both attention conditions.

**Figure 7:**
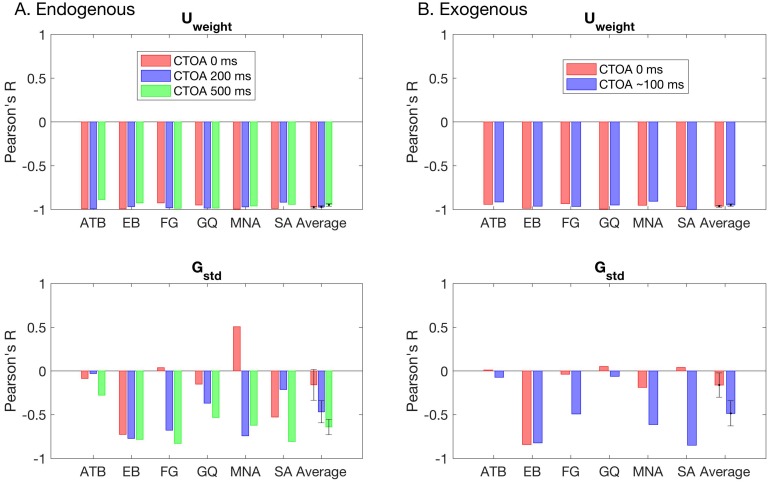
The correlations between masking functions and the GU model parameters for the (**A**) endogenous, and (**B**) exogenous attention conditions. The top row represents the standard deviation of the Gaussian whereas the bottom row represents the weight of the Uniform.

To determine how well the changes in transformed performance are reflected in the model parameters, we carried out a correlation analysis, where we computed the Pearson’s R coefficient between masking functions and each model parameter separately. Figure 7 shows the individual and average correlation coefficients for both attention conditions. Different colors represent different CTOAs. Consistently, we found very strong correlations between the weight of the Uniform and the masking functions in all CTOAs. This suggests that regardless of the level of attentional resources on the target bar, the transformed performance can be closely captured by the changes in the weight of the Uniform term.

## 5. Discussion

The visual system is overwhelmed by an enormous amount of information impinging on the retina. Since the computing resources available to the brain are limited, they must be used efficiently. Spatial attention facilitates this feat by selecting a relevant subset of information and filtering out or suppressing the rest. In other words, it controls the quality and quantity of information transfer from sensory input to VSTM. Visual masking also plays an important role in the transfer of information from sensory memory to VSTM. In fact, many studies on VSTM have used visual masks to control the information available to the observer. However, this approach neglects the possibility of interactions between masking and attention mechanisms. In such studies, interactions effects between VSTM and attention may in fact be confounded with spurious interactions between masking and attention when ceiling and floor effects are present.

Earlier studies on masking and attention relations indeed showed significant interactions between the two [18,19,20,21,22,69,70,73]. In common-onset masking, where the target and mask onsets coincide, but the mask outlasts the target, Enns and Di Lollo [19] showed that attentional benefits due to a spatial pre-cue or reduced set-size strongly depend on mask duration. Similarly, by using metacontrast masks, Tata [22] showed that increasing set size results in an SOA-dependent impairment of performance. However, most of these studies suffered from ceiling/floor effects and possibly other methodological artifacts. For instance, in Tata’s experiments, there was essentially no masking at all for set size one; percentage of correct responses as a function of SOA formed a flat line at around 95%. However, for larger set sizes, they found strong masking effects, and therefore, this led the author to conclude that attention and metacontrast masking interact.

Evidence from recent studies, where ceiling and floor effects were avoided, suggests that mechanisms underlying common-onset masking and attention are indeed independent [23,24,74,75]. Similarly, we sought to determine whether the same relationship holds for metacontrast masking and attention. In a recent study, we varied the number of potential targets in the target display and the SOA to control the attentional load and masking strength, respectively [55] and showed that metacontrast masking and attention operate independently. There are, however, two caveats with this methodology. First, the temporal dynamics of attention and mask interactions cannot be examined by solely manipulating target-display size. Second, since the oriented bars that served as the target was unknown to the observers in the beginning of each trial, they had to attend to the entire target display. Moreover, the target bar was indicated by the onset of a mask. Therefore, the task employed both endogenous and mask-evoked exogenous attention. Differential contributions from endogenous and exogenous attention mechanisms might have obscured a potential interaction between metacontrast masking and attention in our previous study. Finally, since the mask also served as the attentional cue, it was not clear how this dual role might have affected the results. In the present study, we investigated the relationship between metacontrast masking and these two different types of attention separately by using spatial pre-cues that were independent of masks. The task of the observers was again to report the orientation of the cued bar. We kept the set size fixed and varied the CTOA between the pre-cue and the target array and the SOA between the target and mask arrays. We found that for both attention types, the mean error magnitude is affected by CTOA equally at all SOA values. In other words, masking functions underwent vertical shifts with changes in CTOA, indicating that metacontrast masking and attention arise from independent processes. We expressed our data parametrically by using statistical mixture modeling and found that the parameter corresponding to guess rate (i.e., the weight of the Uniform distribution) gave the best account of metacontrast functions. Interestingly, when we further examined interactions at the parametric level, two (three) out of six observers in the endogenous (exogenous) attention condition showed significant interactions between CTOA and the guess rate. Although it was “barely worth mentioning” from a Bayesian statistics point of view [72], individual differences found in model parameters warrant further investigations, especially in the light of recent findings that indicate genetically-based individual variations in metacontrast masking [76,77]. Since we have not genotyped our observers, we cannot generalize our results across all genotypes and we cannot assert whether the individual differences stem from genetic variations.

### 5.1. Implications for Models of Attention

Next, we will discuss whether and how our results can be explained by two prominent models of attention in the literature, namely the Perceptual Template Model (PTM) developed by Lu and Dosher [49], and the Integrated System Model (ISM) developed by Smith and colleagues (Reference [69]—early version, no explicit VSTM layer; Reference [78]—VSTM stage is added; Reference [73] —final version). These models are selected since they also address visual masking and its proposed interactions with attention. In short, PTM can distinguish three attention mechanisms that have distinct signatures on behavioral improvements in perceptual tasks. According to PTM, attention enhances visual stimuli, removes external noise, and reduces multiplicative internal noise. These mechanisms can work in tandem or separately depending on the stimulus configuration and the amount of noise in the stimuli. ISM assumes that attention affects the rate of information transfer from sensory memory to VSTM [79]. Masks either truncate sensory information prematurely before the truncated information is fully transferred to VSTM, or they add noise to the stimulus, which in turn, slows down the rate at which encoded stimulus information becomes available for later stages of processing [73]. Moreover, ISM also assumes that masking and attention mechanisms interact, and hence, predicts larger attentional benefits when a stimulus is masked compared to when it is unmasked. Likewise, the stronger the masking is, the larger the attentional effects will be.

One way by which masking and attention are related in PTM is that the mask adds noise through temporal integration at the stage of the perceptual template, where stimulus enhancement mechanism of attention also operates. Moreover, in a series of studies, Dosher and Lu showed that external noise exclusion is the mechanism underlying endogenous attention effects whereas both external noise exclusion and stimulus enhancement are in play when exogenous attention operates [47,48,49,50]. Therefore, PTM predicts that the amount of external noise added due to the mask will decrease with increasing SOA, hence a Type-A masking function. PTM further predicts that the effect of attention should be large when external noise is large compared to the signal. Hence, it predicts that the effect of attention should be largest at SOA = 0 and should decrease with increasing SOA. These predictions clearly do not hold for our findings. We obtained Type-B masking functions with increasing, rather than decreasing masking effects as SOA increases from zero. Furthermore, we found that the effect of both endogenous and exogenous pre-cues, measured by mean magnitude of errors in orientation judgments, is virtually the same across all SOAs. Based on the Type-B shape of masking functions, one could speculate that, by some unspecified mechanism, the metacontrast mask adds external noise in an SOA-dependent manner, i.e., less noise at very short and long SOAs and more noise at intermediate SOAs where masking is strongest. According to this scenario, an increase in CTOA should lead to larger change in performance at intermediate SOAs compared to short and long SOAs. This is equivalent to a statistical interaction between SOA^2^ and CTOA. As revealed by statistical modeling of the distribution of signed response-errors, rather than just the mean magnitude of errors, the interaction between SOA^2^ and CTOA was evident in the frequency of random guessing behavior for two observers in the endogenous attention condition, and for three observers in the exogenous attention condition. In sum, although the underlying neurophysiological mechanism is unspecified at this time, our finding that there might be modest interactions between metacontrast masking and attention can be explained by PTM. However, as mentioned above, this explanation rests on some unspecified mechanism, according to which the metacontrast mask adds external noise in an SOA-dependent manner.

ISM makes predictions similar to those of PTM. However, as mentioned before, ISM directly incorporates interacting masking and attention mechanisms. For instance, it predicts that there will be no effect of attention in the absence of masks. However, our baseline data, which correspond to no mask conditions (see Figure 3C), show clear effects of attention and we did not find strong evidence in favor of interactions between attention and masking.

### 5.2. Implications for Masking Models

Attention has facilitative and inhibitory effects in almost all perceptual tasks [30,80]. However, many early models of visual masking do not address the effects of attention on masking, and mostly assume that attention and masking are independent processes (e.g., References [5,81,82]). These models can be extended straightforwardly to include attention as an add-on process, which simply reduces the masking strength uniformly across SOAs. Michaels and Turvey’s model [83] also included attention as an independent process, which modulates spatial inhibitory effects in masking. 

At least one theory of visual masking puts more weight on attention [18,19]. In a common onset masking paradigm, Enns and Di Lollo [19] showed that four-dot masks can produce strong masking when the stimuli were viewed peripherally *and* when attention was diffused to more than one spatial location. Enns and Di Lollo interpreted these effects as a result of re-entrant (feedback) higher-level processes contributing to *object substitution*. In summary, interaction between attention and masking is an essential ingredient of the object substitution theory. This prediction was supported by significant interactions found in their study [18,19]. However, as noted, more recent evidence shows that their results suffered from ceiling/floor artifacts, and that common-onset masking and attention do not interact [23,24,75]. Another strong contradiction to the object substitution theory comes from a study by Filmer, Mattingley, and Dux [24]. They found strong common-onset masking effects for attended and *foveated* targets. Consistent with these recent reports, here we showed that when targets are masked, manipulations of the levels of metacontrast and of endogenous/exogenous attention have largely independent effects. In addition, from a theoretical point of view, Francis and Hermens [84] argued that re-entrant processes, a prominent feature of common-onset masking, are not necessary to explain its results, since they can be also captured adequately by feed-forward models of masking. The way they modeled attentional effects was by reducing the intensity of the mask stimulus. However, since changes in target/mask energy ratio generally changes the shape of masking functions [2] and given the aforementioned recent evidence for lack of interactions between common-onset masking and attention, alternative ways of modeling attentional effects may be more appropriate (e.g., Reference [85]; see also, References [86,87]).

It would be interesting to see whether this finding holds when there are varying types of high external noise in the stimuli. For example, a compound mask consisting of noise spatially overlapping the target added to a non-overlapping metacontrast-type stimulus could be used in conjunction with spatial cues to test whether masking and external noise exclusion mechanism of attention also do not interact.

## Figures and Tables

**Table 1:** The target, mask, and cue luminance values in cd/m^2^ (and corresponding Weber contrast values) are listed for each observer in endogenous and exogenous attention conditions. The background luminance was 60 cd/m^2^ for all observers. The results of *t*-tests used to assess whether criteria C1 and C2 are met are also listed for each observer. Note that we used two-sample *t*-tests with unequal variances for testing for criterion C1, and one-sample *t*-tests against chance level (0.5) for testing for criterion C2.

**Endogenous**					
	**Luminance (Contrast)**			**Statistical Criteria**	
**Observer**	**Target**	**Mask**	**Cue**	**C1 (Ceiling)**	**C2 (Floor)**
ATB	*43 (−0.28)*	*15 (−0.75)*	*10 (−0.83)*	t(150.7) = −2.18; *p* = 0.016	t(99) = 4.11; *p* < 0.001
EB	*12.5 (−0.79)*	*30 (−0.5)*	*10 (−0.83)*	t(164.8) = −2.22; *p* = 0.014	t(99) = 6.15; *p* < 0.001
FG	*46 (−0.23)*	*18 (−0.7)*	*10 (−0.83)*	t(145.5) = −2.73; *p* = 0.004	t(99) = 8.27; *p* < 0.001
GQ	*46 (−0.23)*	*0 (−1)*	*10 (−0.83)*	t(141.6) = −2.53; *p* = 0.006	t(99) = 3.92; *p* < 0.001
MNA	*42 (−0.3)*	*20 (−0.67)*	*10 (−0.83)*	t(142.6) = −2.19; *p* = 0.015	t(99) = 5.86; *p* < 0.001
SA	*47 (−0.22)*	*18 (−0.7)*	*10 (−0.83)*	t(138.6) = −2.94; *p* = 0.002	t(99) = 7.72; *p* < 0.001
**Exogenous**					
	**Luminance (Contrast)**			**Statistical Criteria**	
**Observer**	**Target**	**Mask**	**Cue**	**C1 (Ceiling)**	**C2 (Floor)**
ATB	*44 (−0.27)*	*10 (−0.83)*	*30 (−0.5)*	t(167.7) = −2.23; *p* = 0.013	t(99) = 6.26; *p* < 0.001
EB	*40.5 (−0.32)*	*12 (−0.8)*	*30 (−0.5)*	t(181.5) = −1.87; *p* = 0.031	t(99) = 5.24; *p* < 0.001
FG	*46 (−0.23)*	*18 (−0.7)*	*30 (−0.5)*	t(180.6) = −2.5; *p* = 0.007	t(99) = 6.26; *p* < 0.001
GQ	*46.5 (−0.22)*	*6 (−0.9)*	*30 (−0.5)*	t(125.3) = −2.9; *p* = 0.002	t(69) = 4.34; *p* < 0.001
MNA	*43.5 (−0.28)*	*30 (−0.5)*	*30 (−0.5)*	t(137) = −2.34; *p* = 0.01	t(99) = 6.68; *p* < 0.001
SA	*48 (−0.2)*	*30 (−0.5)*	*30 (−0.5)*	t(134.6) = −3.81; *p* < 0.001	t(99) = 3.73; *p* < 0.001

**Table 2:** The regression models used to fit transformed performances and the winning model parameters are listed. The models are sorted based on number of parameters. The models M1, M2, M3, M4, M7, M8, M9, and M14 are the standard linear regression models whereas the remainder of models has quadratic main factors and/or interactions. τ represents SOA (Stimulus Onset Asynchrony) and n represents CTOA (cue-target onset asynchronies). β’s are the coefficients of the models and ε represents the error term.

*ID*	*Regression Model*
*M1*	Y = β_0_ + ε
*M2*	Y = β_0_ + β_1_ τ + ε
*M3*	Y = β_0_ + β_1_ n + ε
*M4*	Y = β_0_ + β_1_ τ n + ε
*M5*	Y = β_0_ + β_1_ τ^2^ + ε
*M6*	Y = β_0_ + β_1_ τ^2^ n + ε
*M7*	Y = β_0_ + β_1_ τ + β_2_ n + ε
*M8*	Y = β_0_ + β_1_ τ + β_2_ τ n + ε
*M9*	Y = β_0_ + β_1_ n + β_2_ τ n + ε
*M10*	Y = β_0_ + β_1_ τ^2^ + β_2_ n + ε
*M11*	Y = β_0_ + β_1_ τ^2^ + β_2_ τ^2^ n + ε
*M12*	Y = β_0_ + β_1_ n + β_2_ τ^2^ n + ε
*M13*	Y = β_0_ + β_1_ τ + β_2_ τ^2^ + ε
*M14*	Y = β_0_ + β_1_ τ + β_2_ n + β_3_ τ n + ε
*M15*	Y = β_0_ + β_1_ τ^2^ + β_2_ n + β_3_ τ^2^ n + ε
***M16***	**Y = β_0_ + β_1_ τ + β_2_ τ^2^ + β_3_ n + ε**
*M17*	Y = β_0_ + β_1_ τ + β_2_ τ^2^ + β_3_ τ n + ε
*M18*	Y = β_0_ + β_1_ τ + β_2_ τ^2^ + β_3_ τ^2^ n + ε
*M19*	Y = β_0_ + β_1_ τ + β_2_ τ^2^ + β_3_ n + β_4_ τ n + ε
*M20*	Y = β_0_ + β_1_ τ + β_2_ τ^2^ + β_3_ n + β_4_ τ^2^ n + ε
*M21*	Y = β_0_ + β_1_ τ + β_2_ τ^2^ + β_3_ n + β_4_ τ n + β_5_ τ^2^ n + ε

**Figure A1:**
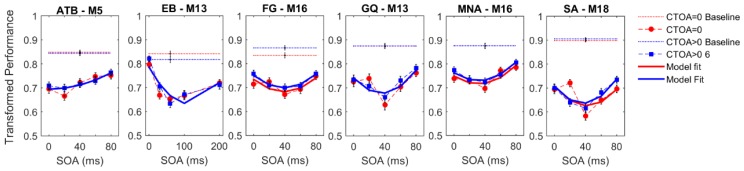
The transformed performance as a function of SOA for invalid trials in the exogenous attention condition for all CTOAs. The horizontal axes represent SOA and the vertical axes represent transformed performance. Red color represents zero CTOA and blue color represents positive CTOA value (ranging from 80 to 120). The dotted horizontal lines indicate baseline performance. The markers and the dashed lines represent empirical data whereas the solid lines show the best-fit regression model. Each panel shows data from a single observer. The best regression model (see Table 2) is given on top of each panel. Error bars represent ±SEM across trials (n = 100).

**Figure A2:**
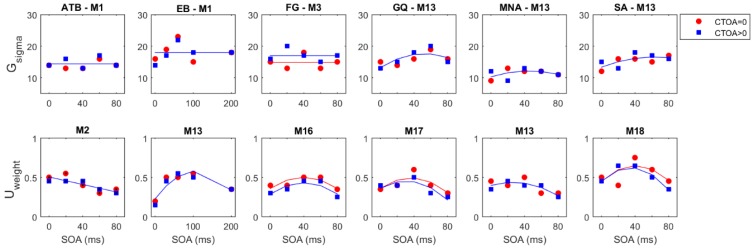
The transformed performance as a function of SOA for invalid trials in the exogenous attention condition for all CTOAs. The horizontal axes represent SOA and the vertical axes represent transformed performance. Red color represents zero CTOA and blue color represents positive CTOA value (ranging from 80 to 120). The dotted horizontal lines indicate baseline performance. The markers and the dashed lines represent empirical data whereas the solid lines show the best-fit regression model. Each panel shows data from a single observer. The best regression model (see Table 2) is given on top of each panel. Error bars represent ± SEM across trials (n = 100).

## Appendix A

As mentioned in the Methods section (Section 2), the validity of the cue was 25% in the exogenous attention condition to make sure that the cue is non-informative and the effect of endogenous attention is minimized or eliminated. Observers completed 1200 valid trials out of roughly 4800 trials. We analyzed the invalid trials in exogenous attention condition separately. Figure A1 shows the experimental results of different SOA and CTOA conditions for all observers. X axes represent SOA and y axes represent transformed performance. Different colors represent different CTOA conditions. The dotted lines show baseline condition for each CTOA value. The markers and dashed lines show the empirical data and the solid lines show the best-fitting regression model (see Table 2). Only two (FG and MNA) out of six observers showed a significant main effect of CTOA. In invalidly cued trials, the effect of attention was much weaker compared to validly cued trials.

We looked at the distribution of signed response-errors of invalid trials in the exogenous attention condition and fitted a hierarchy of statistical models described in Methods section (Section 2). The GU model was the winning model for all observers as in the valid trials presented in the main text. Figure A2 shows the model parameters for the GU model as a function of SOA and CTOA. Red color represents zero CTOA and blue color represents positive CTOA values (ranging from 80 to 120). Markers show data points and solid lines show the best fitting regression models. The effect of attention was not significant on standard deviation of Gaussian except for observer FG, and there is no discernable pattern of changes in standard deviation of Gaussian in the GU model. Although the weight of the Uniform follows an inverted shape of the masking function for most (five out of six) of the observers, the effect of attention was not significant on the weight of the Uniform distribution in GU model except for observer FG. Overall, attention effects are much weaker in the invalidly cued trials in the exogenous attention condition. Therefore, it is not ideal to investigate whether there is an interaction between attention and metacontrast masking in invalid trials since the main effect of attention is absent.

## Appendix B

To show how well the best fitting models fit the data, Table A1 summarizes the R^2^ values for the best-fitting models for all observers and experiments.

**Table A1:** A summary the R^2^ (and Adjusted R^2^) values for the best fitting models for all observers and experiments.

	ATB	EB	FG	GQ	MNA	SA
**Endogenous**	0.82 (0.74)	0.97 (0.95)	0.96 (0.93)	0.90 (0.87)	0.81 (0.75)	0.89 (0.85)
**Exogenous**	0.84 (0.73)	0.90 (0.81)	0.75 (0.54)	0.94 (0.90)	0.85 (0.76)	0.91 (0.85)
